# The Physical History of Various Nations of the Earth, with Special Reference to Their Teeth

**Published:** 1867-11

**Authors:** J. Allen


### ARTICLE IV.

### *The Physical History of Various Nations of the Earth, With Special Reference to their Teeth.*

By Dr. J. Allen.

**Read before the American Dental Association, in Cincinnati, Aug. 2, 1867.**

Having spent some thirty-eight years in Dental practice, I have often been asked these two questions: first, "are not the teeth of the people of this country worse than those of other nations of the world?" And, second, "what is the cause of so many bad teeth in America?" These are two important questions involving the welfare of some thirty millions of inhabitants. In order to answer them satisfactorily, we have found it necessary to examine the physical history of mankind, in order to compare nations with nations in reference to their teeth, taking into consideration their food, habits, customs, climate, etc. etc.

In prosecuting these researches we find there are many nations whose teeth remain sound, even to old age, and it is as rare for them to lose a tooth as it is an eye or a limb. While in this country it is estimated that there are more than twenty millions of teeth lost annually from decay. And yet we find that the same general physical law which provides for the building up and sustaining the human structure, prevails among all nations, and that the divine architect of man has furnished an abundant supply of materials for all parts of the system. The body of man, with all its different parts and organs, is composed of only a few simple materials. These are combined in certain proportions, in order to give strength and utility to the whole structure. These materials are component parts of his food; and although the nutrient substances used by the inhabitants of different parts of the world appear quite dissimilar, yet the food provided for them in various countries possesses the same general nutrient properties and chemical constituents everywhere that are essential for the human organism.

We will now proceed to notice some of the historical evidences which go to establish the fact that the Americans, as a whole, have worse teeth than the inhabitants of other nations. In portions of Europe, where the people, like the Americans, discard a large portion of the mineral element from their food, they also have bad teeth ; but among the Peasantry, and also in those sections where the inhabitants do not change the proportions of the mineral constituents of their food, they have good teeth

But let us turn to the historical accounts of other countries where bolting cloths are not used for this purpose.

In Prichard's Researches into the Physical History of mankind he says : "the Albanians of Lesser Asia live principally on milk, cheese, eggs, olives and vegetables. Sometimes they bake bread, but often eat their corn or maize boiled." Hippocrates says they are very strong and muscular, have oval faces, a ruddy color in their cheeks, a brisk animated eye, a well proportioned mouth, and *fine teeth.* In Central Africa, north of the equator ; Prichard says "the Mandingo tribes have the barbarous custom so common among the Pagans of Africa, of filing their teeth to a point."

In eastern Africa, among the different races of Abyssinians, we have the following description by this eminent author : "Their countenance is full without being puffed, their eyes are beautiful, their mouth of moderate size, their lips thick, their *teeth white, regular, and scarcely projecting.*" Among the races of people inhabiting Nubia and other countries between Abyssinia and Egypt, Burckhardt says : "They are a handsome and bold people of a dark brown complexion, with beautiful eyes and *fine teeth.*" In the western parts of South Africa, comprising the Congo Empire, Proyart, who has graphically described it, says ; "The negroes are well made, very black, with *white teeth* and pleasing countenances." In

Dr. Oldfield's ethnographical researches in the interior of Africa, among the Felatahs, he says: "The color is light brown, features regularly formed, handsome mouth, thin lips, *with teeth as white as ivory.*"

We will now pass into Asia, and there among the mountain tribes of Dekham in India, Dr. Maxwell says: "The Khonds are a dark race of men, straight, well limbed, and free from obesity, which makes them have a tall appearance. Many of the men have a pleasing expression of the countenance. Generally, however, the nose is flattish, the cheek bones high, the face round, the lips and mouth large, displaying *fine teeth.* The country produces rice, and most of the vegetables which are common in Europe." Among the Turkish tribes of Kiptschak, the Tartars of Kasan," says Erman, " are of middle stature and muscular, but not fat. Their heads are of an oval shape, their countenances of fresh complexion, and fine, regular features; their eyes, mostly black, are small and lively; their noses arched, and thin, as well as their lips; their hair is generally dark, and their *teeth strong and white.*" We will now pass to that part of Asia between Hindostan and China, where we find, according to Finlayson, that the Siamese blacken their teeth and redden their mouths with a masticatory of lime, catechu and betel, which gives them a disgusting appearance. Baron Larry, who is well known as an eminent author on physical subjects, says: "The inhabitants of Eastern Arabia are somewhat above the average statue, robust and well formed. Their countenances oval, and copper colored, the forehead broad and elevated, the eyebrows black and bushy, the eye dark, deep-seated and quick, the nose straight and of moderate size, the mouth well-shaped, *the teeth beautiful and white as ivory.*" "In Egypt," the same author says, "the surface of the jaws of the Arabs are of great extent and in a straight or perpendicular line. The alveolar arches are of moderate size, and supplied with *very white and regular teeth,* the canines especially, project but little.'' The Arabs eat little and seldom of animal food.

We will now pass to a group of islands situated in the great Southern Ocean, between the eastern coast of Africa and the western shores of the new or American Continent. This group of islands received from Captain Cook, the name of the Society Islands. Mr. Ellis who spent some six years among the inhabitants of Tahiti as a missionary, had ample opportunity of observation, says: "These people are above the middle stature: in physical power they are inferior to the New Zealanders. The mouth of the Tahitian, he says, is well formed, though the lips are sometimes large, yet never so much so as to resemble those of the African. *The teeth are always entire,* except in extreme old age, and though rather large in some, they *are remarkably white and seldom either discolored or decayed.*''

Mr. Anderson, who visited New Zealand with Captain Cook, says: "The nations do not exceed the common stature of Europeans, and in general are not so well made especially about the limbs. Their color is of a different cast, varying from a pretty deep black to a yellowish or orange tinge, and their features are also various, some resembling Europeans. Their faces are round, with full lips, their eyes large, hair black, straight and strong. *Their teeth are commonly broad, white and well set.*'' Another writer, Captain Fitzroy, in describing the people of New Zealand, where he speaks of their teeth, says: "They are like those of the Tucgians, and, at the first glance, remind one of those of a horse. Either they are all worn down, in old persons, canine, cutting teeth, and grinders, to an uniform height, so that their interior texture is quite exposed, or they are of a peculiar structure,'' undoubtedly the former''. The natives who live near the hot, sulphurous waters on the borders of the lake of the Roturna, have the enamel of their teeth, especially their front teeth, yellow, although this does not impair their soundness, and is the effect, probably, of the corroding qualities of the thermal waters. To the eastward of the Society Islands, in the South Pacific, are the Gambier Islands. They are inhabited by a people fairer than the Sandwich Islanders. The average height of the men is about that of Englishmen, but they are not so robust. In their muscles there is a flabbiness, and in the old men a laxity of integument which allows their skin to hang in folds on different parts of their body. They have an Asiatic countenance, the teeth in the fourth class especially are not remarkable for evenness or whiteness, and seem to fall out at an early period. With reference to these physical characteristics, Dr. Pritchard says: "Two causes may be assigned: the nature of their food and their indolent habits."

We will now pass to Easter Island, which is situated perhaps the most remote from the great continents of all inhabited islands on the globe. Captain Beechey has given the following physical account of the inhabitants. He says "They are a fine race of people, especially the women. They have oval countenances, regular features, a high and smooth forehead, black eyes and *fine teeth.*"

Next, let us take a view of the Sanwan group of islands, situated also on the Pacific ocean, in latitude thirteen and fourteen degrees The inhabitants of these islands are strong, vigorous, and well proportioned. Their features are all referable to a common type. This type is thus minutely described: "The nose is short and wide at the base; the eyes are black, and often large and bright, the forehead narrow and high, the mouth large and well filled with *white and strong teeth.*" These islands abound in pigs, dogs, fowls, birds and fish, and likewise in cocoa nuts, guava, banian trees and sugar canes. Belonging to another group, in the same ocean, are the Tarawan Islands. The people of this group differ from those above described. They are of middle size, their color is dark copper, their hair is fine, black and glossy, the nose slightly aquiline, the mouth is large, with full lips and *sound teeth.*

Vanikoro, another group of these islands, is also in this great ocean. The sea coast is inhabited by a black race, who cultivate the taro, iguamas, bananas and the kava. "The inhabitants," says Dr. Urville, "belong to the black race of the great ocean approaching to that of proper negroes. They are generally small, their countenance has a singular resemblance to the ourang-outang, the eyes are large, and deeply set, resembling in form and color those of the negro. The lips are large, the chin small, the hair crisp. The use of the betel root destroys their teeth, and gives them a red tinge round the mouth. The women are horribly ugly, the old men are bald." Next we will proceed to the Archipelago, of the Fiji or Fejee Islands, which lie to the eastward of those above named, and are situated between fifteen and nineteen degrees of south latitude. This a large group of islands, many of which are inhabited. The largest of this group is called the Great Viti. The people of this island are called Vitians. They are tall, well made, active and muscular. Their faces are broad, nose large and flat, large mouths, thick lips, and *sound, white teeth.*

(To be Continued.)

